# Outcome of acute pancreatitis in octogenarians: A retrospective study

**DOI:** 10.1002/jgh3.12279

**Published:** 2019-11-14

**Authors:** Davide Di Mauro, Chinthaka N Wijesurendere, Andrea Attanasio, Claudia A M Fulgenzi, Iyad Elkhuffash, Edoardo Ricciardi, Shahjehan Wajed, Antonio Manzelli

**Affiliations:** ^1^ Department of Upper GI Surgery Royal Devon and Exeter NHS Foundation Trust Exeter UK; ^2^ Medical School University of Exeter Exeter UK

**Keywords:** acute pancreatitis, clinical outcome, octogenarians

## Abstract

**Background and Aim:**

Acute pancreatitis (AP) is a common disease, but data on outcomes in octogenarians are scarce in the literature. The aim of this study is to analyze results from patients aged 80 years old and over who were treated for AP at a single center.

**Methods:**

Patients aged 80 years and older diagnosed with AP from April 2010 to October 2015 were considered. Demographics, American Society of Anesthesiologists (ASA) score, Charlson Comorbidity Index (CCI), serum biochemistry at 24 and 48 h after admission, and revised Atlanta severity score were analyzed and correlated with hospital mortality rate and length of stay using the multiple regression and Kaplan–Meier tests.

**Results:**

A total of 100 consecutive patients were included in the study. There were 52 women, and the mean age was 87.5 years (range 80–95). Gallstones were the most common cause of AP (69.7%). The ASA score was ≥III in 51 patients. Eight patients had severe, disease and all of them died in hospital. A CCI > 4 was associated with higher disease severity and mortality (*P* < 0.00001). The median hospital stay was 9 days (range 1–59). Longer hospital stay was associated with serum C‐reactive protein ≥242 mg/L (*P* = 0.01) and serum albumin ≤30 g/L (*P* = 0.01) at 48 h. Over a 5‐year period, 22% of patients were readmitted to hospital with recurrent AP. Gallstones were the main cause of disease (63.6%).

**Conclusions:**

AP in octogenarians has low mortality. Higher death rate is associated with disease severity. In the presence of gallstone disease, cholecystectomy is recommended whenever possible as the risk of disease recurrence is significant.

## Graphical abstract

Acute pancreatitis in octogenarians has low mortality. Gallstones are the main cause of disease, and cholecystectomy is recommended whenever possible as the risk of disease recurrence is significant.

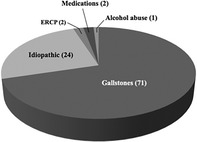

## Introduction

Acute pancreatitis (AP) represents a common clinical scenario, and in the United States, the annual incidence in the adult general population is 13–45/100.000.^1^ The disease occurs more frequently in the fifth decade of life, and gallstones represent the main cause in 35–65% of cases; alcohol abuse accounts for 22%, and in up to 25% of patients, no etiology is identified, and AP is defined as idiopathic.^2^ It is estimated that, in octogenarians, the disease occurs in up to 5/100 000.^3^ In this group, gallstones represent the most common cause, followed by idiopathic and iatrogenic etiologies.^4^ Due to the aging of the general population in developed countries, increased numbers of elderly and frail patients suffering from AP are anticipated.^5^

In the majority of patients, the disease presents as a mild, self‐limiting condition; in such a case, the overall mortality rate is 7%.^6^ AP develops into severe disease in 20% of cases, with mortality rates of up to 15–20%.^7^, ^8^ Outcomes of AP in the elderly seem to be worse than in younger people as higher mortality rates and longer hospital stay had been reported.^3^, ^5^, ^9^ However, most of the published series includes patients aged 65 years and older, with little focus on the very elderly. The aim of the study is to analyze data from a single center on the outcome of AP in patients aged 80 years and older.

## Methods

All patients aged 80 years and older admitted with AP from April 2010 to October 2015 were considered for the study. At our institution, AP is diagnosed in the presence of at least two of the following criteria^10^: abdominal pain, serum amylase >300 IU/L, and findings of AP on radiologic imaging. Exclusion criteria were the presence of periampullary tumor.

Patient's demographics, American Society of Anesthesiologists (ASA) score, cause of disease, Charlson Comorbidity Index (CCI),^11^ serum biochemistry, revised Atlanta severity score,^12^ rate of pancreatic necrosis, mortality, length of hospital stay, and readmission rate were retrospectively analyzed. Mortality was considered when occurring during the same hospital admission.

For statistical analysis, the Chi‐square test was used to evaluate differences in frequencies, while continuous variables were analyzed with the Student's *t*‐test.

Multiple linear regression was applied to test if patients' characteristics were predictors of the mortality rate and length of hospital stay.

The Kaplan–Meier test was used for survival analysis. Two‐tailed *P* values were used and were considered significant if <0.05.

At our institution, AP is diagnosed in the presence of at least two of the following criteria [6]: abdominal pain, serum amylase >300 IU/L, and findings of AP on radiologic imaging. Unless a history of gallstones is evident, all the patients have an abdominal ultrasound scan (USS) of the liver in the first 48 h after admission to check for the presence of gallstones. A computerized tomography (CT) scan of the abdomen is considered in case of clinical deterioration, suspected complications of AP (pseudocyst formation, hemorrhage, infected necrosis), intra‐abdominal sepsis, or to rule out other causes of abdominal symptoms. A magnetic resonance cholangiopancreatography (MRCP) is performed in case of jaundice and elevated serum bilirubin or dilated intra‐ and extrahepatic ducts on USS.

The initial treatment consists of analgesia—intravenous opioids and paracetamol, Ringer's lactate infusion, and oxygen mask depending on the respiratory function; a nasogastric tube is positioned in case of profuse vomiting or if there is evidence of gastric dilatation on examination or on radiologic imaging.

Early oral feeding is encouraged; a nasojejunal feeding tube is inserted in those patients who cannot tolerate or are unable to undergo oral feeding due to poor general conditions.

The infusion rate of intravenous crystalloids aims to achieve a urine output of at least 0.5 mL/kg/h; therefore, it is adjusted based on patients' tolerance, response, and presence of cardiovascular and/or renal dysfunction.

Patients with confirmed diagnosis of common bile duct stones are considered for an endoscopic retrograde cholangiopancreatography (ERCP) during the same admission. Laparoscopic cholecystectomy (LC) is offered during the same admission or electively according to their general conditions and suitability for general anesthesia and surgery.

## Results

**Figure 1:**
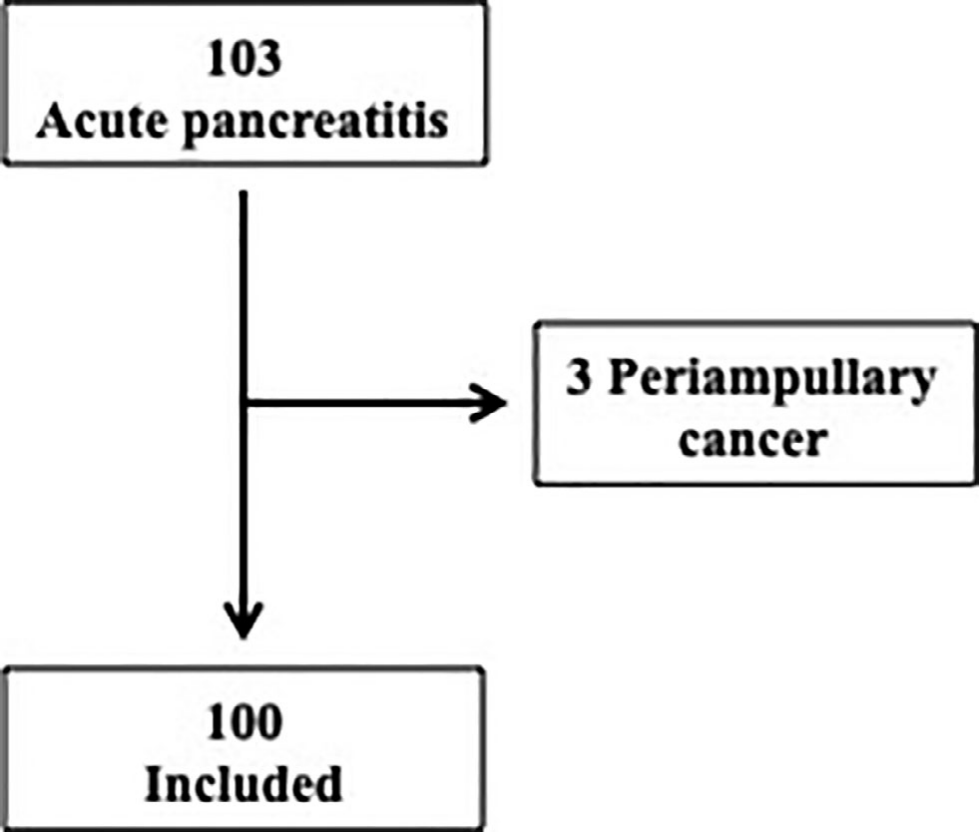
Inclusion and exclusion criteria.

**Figure 2:**
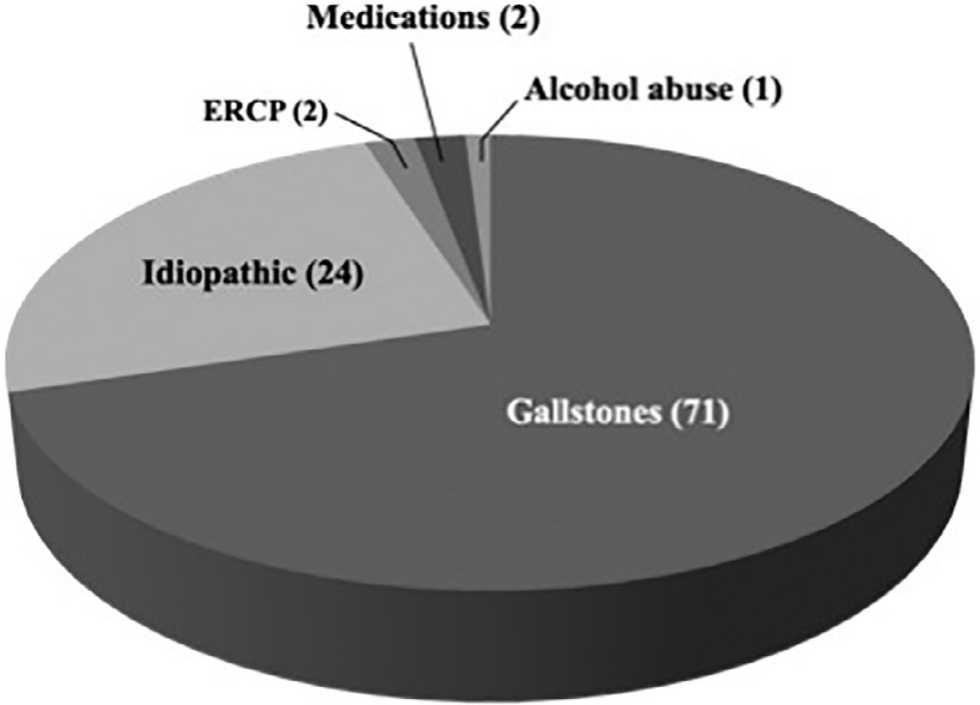
Causes of acute pancreatitis. ERCP, endoscopic retrograde cholangiopancreatography.

Among 103 consecutive patients with diagnosis of AP, 3 were excluded due to the presence of a periampullary tumor. Therefore, 100 patients were included in the study (Fig. 1). There were 52 women; the mean age was 87.5 years (range 80–95). Gallstones were the main cause of disease (Fig. 2). Twenty‐two patients had recurrent AP, which was gallstones‐related in 14 (63.6%) and idiopathic in 8 (36.4%).

Eighty‐seven patients had comorbidities, including hypertension (40), type II diabetes mellitus (17), ischemic heart disease (14), chronic kidney disease (11), and chronic obstructive pulmonary disease (5). An ASA score ≥ III was recorded in 51 cases; 52 patients had CCI > 4 (Table 1).

**Table 1:** Patients' demographics

Parameter	Number
Mean age (range)	87.5 (80–95)
Gender	W 52, M 48
ASA	
I	12
II	37
III	16
IV	35
Median CCI (range)	
CCI 4	48
CCI 5–8	47
CCI 9–11	5

ASA, American Society of Anesthesiologists; CCI, Charlson Comorbidity Index.

Upon admission, high C‐reactive protein (CRP) level was recorded in 79% cases, hypoalbuminemia in 10%. Forty‐eight hours later, CRP increased by 275% in 98 patients (*P* = 0.0001), and albumin level dropped in 42 (*P* = not significant [NS]) (Table 2). According to the Atlanta score, eight patients had severe pancreatitis: one developed acute respiratory distress syndrome (ARDS), and seven had multiorgan failure, consisting of the association of respiratory and renal failure in five cases, cardiac and renal failure in one, and general demise in one (Table 3). CCI > 4 was associated with severe disease (*P* < 0.00001). Of the patients, five were admitted to the intensive care unit (ICU).

**Table 2:** Serum biochemistry results

Parameter	Upon admission mean (range)	At 48 h mean (range)	Normal range	*P*
Creatinine (μmol/L)	137.4 (92–156)	136.4 (85–208)	44–80	NS
eGFR (mL/min/1.73 m^2^)	44.5 (7–89 mL)	55.5 (6–90)	90–120	NS
CRP (mg/L)	80.2 (1–348)	220.1 (3–485)	0–5	0.0001
Albumin (g/L)	40.4 (10–50)	32.8 (19–43)	35–50	NS

CRP, C‐reactive protein; eGFR, estimated glomerular filtrate rate; NS, not significant.

**Table 3:** Patients' clinical outcomes

Parameter	Number
Atlanta severity score	
Mild	18
Moderate	74
Severe	8
Causes of disease severity	
Respiratory failure	1
Multiorgan failure	7
Pancreatic necrosis on CT scan	7
Percutaneous drainage	1
ERCP	8
LC during the same admission	2
Mortality	8
Median length of stay, days (range)	9 (1–59)
Number of readmissions	25

CT, computed tomography; ERCP, endoscopic retrograde cholangiopancreatography; LC, laparoscopic cholecystectomy.

[Corrections added on 20 January 2020, after first online publication: Numbers for ‘Moderate’ and ‘Severe’ on the Atlanta severity score have been revised. The parameter ‘Renal failure’ has been amended to ‘Respiratory failure’ and the corresponding number has been corrected.]

An abdominal CT scan was performed in 65 patients. It detected pancreatic necrosis in seven (10.8%) cases, six of which were men (*P* = NS). Infected pancreatic necrosis was diagnosed in one patient, who was treated with percutaneous drainage. Among those with gallstone AP, eight (11.3%) patients had common bile duct stones, which were detected by CT (1) and MRCP (7). All of them underwent ERCP, which was successful in four. In the remaining four patients, stones were not detected.

Two (2.8%) patients underwent LC during the same admission. Five (7.1%) refused the procedure; 40 (56.3%) were deemed too frail to undergo either general anesthesia or surgery.

All the eight patients with severe AP died during the hospital admission. Six (75%) had idiopathic disease, and two (25%) had gallstones.

**Figure 3:**
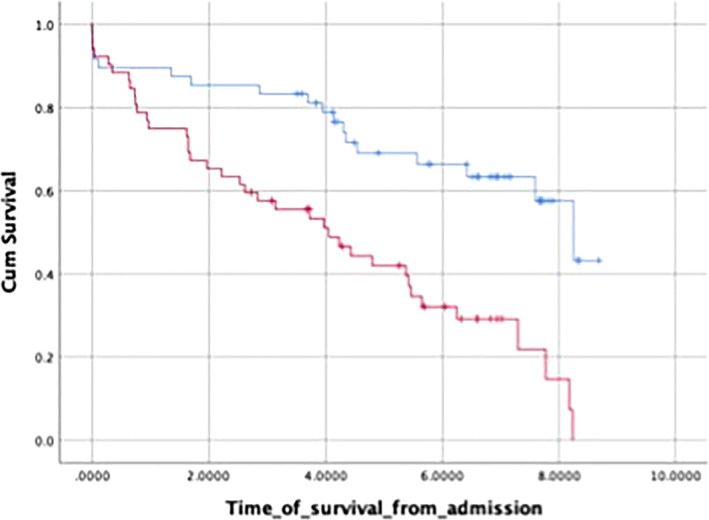
Charlson Comorbidity Index and mortality. (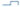), 4; (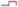), >4; (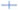), 4‐censored; (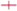) >4‐censored. CCI, Charlson Comorbidity Index.

Patients' gender, ASA score, serum CRP, and albumin on admission and at 48 h were not associated with higher mortality rate (*P* = NS), but CCI > 4 was (*P* < 0.0001; Fig. 3).

The median hospital stay was 9 days (range 1–59). Longer stay was associated with serum CRP ≥242 mg/L and serum albumin ≤30 g/L at 48 h. CRP cut‐off level at 242 mg/L showed 70% sensitivity (95% confidence interval [CI] 55–82%) and 75% specificity (95% CI 62–85%); area under curve (AUC) 0.76 (95% CI 0.64–0.87) (*P* = 0.01). Serum albumin cut‐off at 30 g/L showed a sensitivity of 48% (95% CI 34–63%) and a specificity of 77% (95% CI 63–87%); AUC 0.63 (95% CI 0.51–0.76) (*P* = 0.01). There was no correlation between CCI > 4 score and hospital stay (*P* = NS).

Among those with gallstones, 24 (33.8%) underwent elective LC. Over a 5‐year period, 25% patients were readmitted in hospital with recurrent AP. Sixteen (64%) had gallstones AP, and nine (36%) had idiopathic disease. Three had cholecystectomy before, one underwent LC during the same admission, six underwent elective cholecystectomy, and six were considered not fit for surgery.

## Discussion

AP is common, but unfortunately, a few studies only have been published on the disease outcome in octogenarians. Patients aged 80 years and older represent an unique group as their response to treatment may differ from that of young adults; moreover, the presence of comorbidities and the frail status, which are often associated, make treatment more challenging. This can explain the lack of data on such a group of patients, who are considered less representative of the general population.^9^ In general, the cut‐off definition for older adults is not homogeneous, and a comparison with our cohort of octogenarians is difficult.^13^

Gallstones and idiopathic disease had been reported as the main causes of AP in the elderly,^3^, ^4^, ^14^, ^15^ and our results confirm that. Older age is associated with a higher prevalence of comorbidities, frailty, and reduced cardiorespiratory reserve, all of which account for a higher incidence of AP‐related multiorgan failure and mortality.^13^, ^16^ Interestingly, in our study, mortality was only 8%, and multiorgan failure was the main cause of death.^2^, ^4^, ^9^, ^13^, ^14^, ^15^, ^16^, ^17^ Moreover, mortality in our series was far lower than that of the general population.^7^, ^8^ Perhaps the reduced inflammatory response due to the relative immunosuppression in the elderly^18^ may account for a less common development of severe disease. The incidence of local complications of AP occurs more often in younger patients^17^; in our study, pancreatic necrosis was detected in 7 of 65 patients who underwent CT scan, and percutaneous drainage of infected necrosis was required in 1 case only. In light of such a result, it seems that CT scan is not required in the majority of patients and should only be conducted—particularly in those with renal dysfunction—unless an invasive intervention (i.e. percutaneous drainage) is planned.

Of the eight patients who underwent ERCP, only four had common bile duct stones (CBDS). In the remaining four, the stones passed out spontaneously. Therefore, invasive procedures such as the ERCP should be planned carefully in the elderly, and indications should be periodically evaluated.

Roulin *et al*.^19^ found that patients aged 70 years and older had a significantly longer hospital stay (median 11 days *vs* 7 days) than younger patients. In the present study, the median length of hospital stay was 9 days.

Our figures show that one in four patients developed recurrent AP within 5 years. Gallstones remained the most common cause of disease in two thirds of the cases, and the majority of patients did not have cholecystectomy. As it is known that cholecystectomy reduces the recurrence rate of gallstones,^20^, ^21^, ^22^, ^23^ we believe that may partially explain our results. All of the patients who underwent LC had an uneventful postoperative course. These figures are in line with the literature, which supports LC in octogenarians.^24^

In conclusion, the present study shows that AP in patients aged 80 years and older has a low mortality rate. Comorbid conditions, if recognized and managed appropriately, can be effectively tackled without contributing to higher mortality. The use of the abdominal CT scan and ERCP should be planned carefully on a case‐by‐case basis. In case of gallstone AP, cholecystectomy is recommended whenever possible as the risk of disease recurrence is significant.

This study is limited by its retrospective nature, small sample size, and absence of a control group. Moreover, the lack of adequate follow up prevents us from evaluating the incidence of late complications, such as pancreatic pseudocyst or organ exocrine and endocrine insufficiency. Larger prospective trials are necessary to draw more definitive conclusions.
